# Supplementary material for: Qualitative analysis of a remote monitoring intervention for managing heart failure
Source: BMC Cardiovasc Disord. 2023 Sep 7;23:440. doi: 10.1186/s12872-023-03456-9 (PMC10486103; doi:10.1186/s12872-023-03456-9)
Supplement: Supplementary file 1 — Additional file 1. Interview protocols for patients and clinicians are enclosed. [file 12872_2023_3456_MOESM1_ESM.docx]

Appendices: Interview Protocols

CHF Empower Program Patient Interview Protocol

Interviewee:

Interviewer:

Date:

Thank you for agreeing to speak with me today about your experience in the Empower program. This interview is part of a grant project funded by the National Institutes of Health focused on reducing hospital readmission rates for patients with congestive heart failure using remote monitoring devices for monitoring. This interview, along with other activities, will assist us in learning more about how patients felt about the program so we can improve it in the future.  The questions asked are divided into a few different topic areas.

The principal investigator for this project is Dr. Kevin Volpp. If you have any questions or concerns about the project, please call 215-746-4010.

I will treat everything you tell me as confidential in the sense that I will not share it with anyone in a way that identifies you.

This interview will take approximately 45 minutes. Your participation in this interview is strictly voluntary, and you may end this interview at any time. You will be sent $50 for your participation in the interview. Do you have any questions before we begin? Do you agree to participate? Fantastic! I am starting the recorder now.

**Initially, I want to get a sense about your experience in the EMPOWER program.**

1. Overall, how did you feel about the program?
2. What did you like about the program?
3. What did you think could be better about the program?
4. Why did you agree to participate in this project? (Did you think it was worth it? Would you do it again if you could?)
5. Was a family member involved with you during the enrollment process (information, consent, hub setup)? (Did they encourage you to participate?)
6. Do you think being in this program helped you monitor your heart condition? How?
7. Do you think this program helped your doctors monitor your heart condition? How?
8. Were you in touch with your doctor more when you were in the program -or less?
9. If readmitted: Do you think this project had any impact on you having to go back into the hospital? (How? Do you think you would have been at the hospital more if you weren’t on the program?)

If not readmitted: Do you think this project helped you stay out of the hospital while you were enrolled? (Why? Why not?) Do you think you would have had to go back to the hospital more without this program?

**Thank you for that information. Next, I want to ask you some questions about the scale and pill bottle you were given for the project.**

1. Prior to this program, were you weighing yourself regularly?
2. Prior to this program, how did you keep track of your medication?
3. How easy was the scale to use? (Did you have any problems with the scale sending your weights in? Did you need help using the scale?)
4. How easy was the pill bottle to use? (Did you have any problems with the bottle sending your information in? Did you need help using the bottle?)
5. Did you ever open the pill bottle and not take the medicine to turn off the light or sound? (If yes, how often? A few times a week, once a week, not often, etc.)
6. How easy was the hub/2NET Scale Transmitter you received to set up and use? (If problematic, did that impact how you felt about the program?)
7. Did anyone like a friend or family member help you remember to weigh yourself or open your pill bottle while you were in the program?
8. About how much time did you spend every day weighing yourself and opening the pill bottle while you were in the program? (2 minutes? 5 minutes? 10 minutes?)
9. Did you experience any changes in your life while you were enrolled in the program (ie. change of job, move, relationship, etc.)
   1. If yes, did those changes impact your ability to weigh yourself or open your pill bottle every day? How?

**That is very helpful! I want to ask you some questions about how you got your medicine that you took while you were participating in the project.** (NOTE – this is for when they were community dwelling, not in the hospital)

1. How did you get your medication (mail, pharmacy, etc.)? (Did someone get it for you?)
2. Did you have any challenges getting your medication (transportation, cost, pharmacy issues, support system away, etc.? Did those challenges make it harder for you to stay on track with the program?)

**Now, I am going to ask you some questions about the lottery that was part of the EMPOWER program.**

1. Did you win any money in the lottery?
   1. If yes, did you think the amount you won was enough for the time you spent in this program?
2. What did you think about the lottery system? (Did you find the lottery confusing?)
3. Did the lottery make you more likely to weigh yourself and open the pill bottle every day?

**Finally, I want to ask you a few questions about the inspirational messages we sent you during the program.**

1. Did you receive the messages giving you tips for staying healthy?
2. Did you like the messages? (What did you like about them? If no, what didn’t you like?)
3. Do you think the messages helped you stay on track in the program?

**Thank you for taking the time to speak with me today. Your input has been extremely helpful. You will receive your incentive check in the mail the same way you received your lottery payments. Please feel free to contact me with any additional thoughts or questions.**

CHF Empower Provider Interview Protocol and Interview Guide

Interviewee:

Interviewer:

Date:

Thank you for agreeing to speak with me today about your experience with the Empower program. To remind you, this interview is part of the EMPOWER study funded by the National Institutes of Health that tests ways to reduce hospital readmissions for Penn Medicine heart failure patients using remote monitoring devices to track weights and their adherence to a diuretic. The EMPOWER program monitored your heart failure patients at home and alerts were sent into PennChart if your patient had a weight gain of 3 pounds in 24 hours or 5 pounds in 72 hours. These alerts also included information about any symptoms the patient may have been experiencing. This interview, along with other activities, will assist us in learning more about how providers felt about the program so we can improve it in the future.  The questions asked are divided into a few different topic areas. You will be sent a $25 Amazon gift card for your participation.

The principal investigators for this project are Dr. David Asch and Dr. Kevin Volpp and the clinical investigator is Dr. Lee Goldberg. If you have any questions or concerns about the project, please call 215-746-4010.

I will treat everything you tell me as confidential in the sense that I will not share it with anyone in a way that identifies you. All interviews will be taped and ADA Transcription Service will transcribe them. The Senior Qualitative Research Scientist will assist with initial coding and oversee the development of a codebook. Two research coordinators will code each of the interviews. Two research coordinators will code each of the interviews. Codes will be compared at the completion of each set of coding, and any disparities in coding will be discussed and adjudicated by the Research Scientist.

This interview will take approximately 15-30 minutes. Your participation in this interview is strictly voluntary, and you may end this interview at any time. Do you have any questions before we begin?

1. Initially, I would like you to describe how you felt about the program overall.
2. Your team received weight-gain alert notifications from EMPOWER through PennChart for patients enrolled in the program. Please describe how your care team handled an alert from first opening the result to it being resolved.

Probes:

- Who received/managed the alerts?
- Please describe how this fit into your office workflow?
- How much time did the process take?

1. Overall, can you describe how actionable the alerts were?
   - How useful was the information included in the alerts?
   - Were you able to do something about them?
   - Please describe any barriers to managing the alerts (technology problems, communication processes, workflow).
   - Please describe any barriers in managing the patients for whom you received alerts (adherence issues, communication concerns, etc.).
2. Please describe the criteria used to determine how your team responded to alerts.
   - How was it determined whether to respond/call the patient?
   - How did your care team determine what actions to take about patient weight gain and symptoms? (eg. Change diuretic, order lab results, send to ED)
3. Please describe any technical difficulties you faced with the alerts.
   1. Were they sent to the right provider?
4. Did you think the alerts reflected accurate changes in patient health?
   - How did the alerts impact the process of care for patients enrolled in the study?
   - Please describe any ways in which the program impacted your interactions with the patients who were enrolled (eg. Change in diuretics, number of appointments, sent to ED, patients’ relations, e.g., patients happy or unhappy at being contacted).
   - Were there any differences between how you cared for those patients who were enrolled in EMPOWER versus those who were not?
5. Do you recall reviewing PennChart flow sheets of patient weights during the program (for patients who were enrolled)?
   - If yes, please describe how you used them to care for your patients
   - If no, why not?
6. Reflecting on the program overall, what did you like most about the program?
7. What did you think could be better about the program?
8. What challenges did you and your staff (attending, for staff interviews) experience with the program?
9. Would you recommend your patients enroll in a similar program if it was offered in the future? Why or why not?
   - Are there patient characteristics that you think would lead to more or less success in the program?
   - What characteristics may be associated with success based on your experience?
10. We have identified XXX as the staff person in your office who responded to alerts and would be knowledgeable about the EMPOWER program. We plan to invite them for an interview as well. Is this the correct person? If not, can you suggest someone who may be familiar with responding to alerts and would be able to interview with us?

**Thank you for taking the time to speak with me today. Your input has been extremely helpful.**
